# Application of Nanomaterials in Biomedical Imaging and Cancer Therapy II

**DOI:** 10.3390/nano14201627

**Published:** 2024-10-11

**Authors:** James C. L. Chow

**Affiliations:** 1 Radiation Medicine Program, Princess Margaret Cancer Centre, University Health Network, Toronto, ON M5G 1X6, Canada; james.chow@uhn.ca; 2 Department of Radiation Oncology, University of Toronto, Toronto, ON M5T 1P5, Canada

Following the successful publication of the first edition of our Special Issue entitled “Application of Nanomaterials in Biomedical Imaging and Cancer Therapy” [1], we are pleased to present this second edition, which continues to explore cutting-edge advances in the application of nanomaterials for cancer imaging and therapy. Nanotechnology has emerged as a transformative tool in oncology, offering novel solutions for diagnosis, treatment, and theranostics [2]. In this edition, we focus on the integration of nanoparticles in cancer research, addressing key challenges such as treatment specificity, overcoming biological barriers, and enhancing the effectiveness of traditional therapies. The selected studies provide valuable insights into the development of multifunctional nanocomposites, the design of nanoparticle-based drug delivery systems, and innovations in imaging modalities and radiotherapy dose enhancement. This Special Issue aims to advance our understanding of how nanomaterials can be harnessed to improve cancer treatment outcomes and pave the way for clinical translation, while addressing challenges such as biocompatibility, stability, and safety. We hope this collection serves as a valuable resource for researchers and clinicians alike, pushing the frontiers of nanotechnology in cancer care.

Studies on nanoparticle-based imaging and therapeutic applications present a wide range of innovative approaches. Carlton et al. [3] introduce a clinically translatable protocol using Magnetic Particle Imaging (MPI) to guide thermal simulations for Magnetic Particle Hyperthermia (MPH), enhancing treatment planning accuracy. Thomas et al. [4] demonstrate the efficacy of magnet-guided liposomal nanoparticles loaded with temozolomide and ferucarbotran in glioma treatment, overcoming blood–brain barrier challenges while improving imaging and therapy. Petronek et al. [5] further explore MR-based nanotheranostics by using ferumoxytol and pharmacological ascorbate (AscH−) for glioblastoma treatment, highlighting increased toxicity when ferumoxytol is internalized in cancer cells.

Nanoparticles are also employed to enhance imaging techniques. Ostruszka et al. [6] develop a green synthesis method for magneto-luminescent bimetallic nanocomposites (AuNCs-BSA-SPIONs) as dual imaging agents, combining luminescence and MRI contrast for potential clinical use. Wenzel et al. [7] describe the radiofluorination of an amphiphilic teroligomer for stabilizing siRNA-loaded calcium phosphate nanoparticles, enabling PET imaging of brain tumors and providing a tool for tracking nanoparticle distribution.

Theranostic applications in cancer therapy are highlighted by Ruiz-Robles et al. [8], who report the synthesis and characterization of CdTe quantum dots for precise monoclonal antibody and biomarker testing in cellular labeling. Fuentealba et al. [9] present an optimized method for evaluating Gd-nanoparticle dose enhancement in electronic brachytherapy, emphasizing the importance of K-edge interactions in enhancing radiation dose.

Sadiq et al. [10] take a dosimetric approach, using Monte Carlo simulations to study the impact of bone scatter on dose enhancement in nanoparticle-enhanced orthovoltage radiotherapy. Their findings indicate significant underestimation of dose enhancement when bone presence is neglected, particularly at higher nanoparticle concentrations.

From a materials science perspective, Patamia et al. [11] combine halloysite nanotubes with kojic acid to create an antibacterial nanomaterial capable of drug delivery, demonstrating a bio-based approach to antimicrobial cancer therapy. Marforio et al. [12] focus on overcoming the hydrophobicity of carboranes for Boron Neutron Capture Therapy (BNCT) by utilizing blood transport proteins as carriers.

In the realm of reviews, Figueiredo et al. [13] examine metal–polymer nanoconjugates in cancer imaging and therapy, noting that while metallic nanoparticles have unique properties, combining them with polymers enhances biocompatibility, stability, and tumor specificity. Lu et al. [14] review nanoparticle-based therapies targeting the tumor microenvironment (TME) in hepatocellular carcinoma (HCC), addressing the challenges of short drug retention and providing insights into the future of TME-targeting nanomedicine. Siddique et al. [15] highlight advances in functionalized nanoparticles for cancer theranostics, focusing on MRI-guided therapies and photothermal treatments, and discussing their potential to revolutionize personalized cancer care.

The studies presented in this Special Issue underscore significant advancements in the use of nanomaterials for cancer imaging, therapy, and theranostics, offering innovative solutions for more personalized and targeted treatments. These contributions highlight the development of dual-functional nanocomposites for improved imaging and treatment precision, the integration of nanoparticles to overcome challenges like drug retention and the blood–brain barrier, and the critical role of accurate dosimetric planning in radiotherapy. Moreover, the reviews on metal–polymer nanoconjugates, tumor-microenvironment-targeting nanoparticles, and functionalized nanoparticles for theranostics emphasize the potential of these nanomaterials to enhance therapeutic specificity, biocompatibility, and multifunctionality. Together, these findings pave the way for future clinical applications of nanotechnology in cancer care, while addressing ongoing challenges like toxicity, stability, and effective translation to clinical settings.

## Data Availability

Not applicable.
